# Correction: Yang et al. Growing Carbon Nanotubes In Situ Surrounding Carbon Fiber Surface via Chemical Vapor Deposition to Reinforce Flexural Strength of Carbon Fiber Composites. *Polymers* 2023, *15*, 2309

**DOI:** 10.3390/polym15143032

**Published:** 2023-07-13

**Authors:** Guangming Yang, Fei Cheng, Shihao Zuo, Jinheng Zhang, Yang Xu, Yunsen Hu, Xiaozhi Hu

**Affiliations:** 1 School of Materials and Chemistry, Southwest University of Science and Technology, Mianyang 621010, Chinajhzhang2000@163.com (J.Z.); yxu2001@126.com (Y.X.); 2 Engineering Research Center of Biomass Materials, Ministry of Education, Southwest University of Science and Technology, Mianyang 621010, China; 3 School of Mechanical Engineering, Jiangsu University of Science and Technology, Zhenjiang 212003, China; yshu1995@126.com; 4 Department of Mechanical Engineering, University of Western Australia, Perth, WA 6009, Australia

In the original publication [1], ref. [36] was not cited in the caption of Figures 3 and 4.

The citation has now been inserted in the caption of Figure 3 and should read: SEM images of CF: (**a**,**b**) untreated carbon fibers, (**d**,**e**) single carbon fiber after growing VACNTs via CVD [36], (**c**,**f**) corresponding microscopic model diagrams before and after growing VACNTs, respectively. Reprinted with permission from Elsevier 2023 [36].

The citation has now been inserted in the caption of Figure 4 and should read: BET analysis results [36] of CF with growing VACNTs in situ: (**a**) adsorption and desorption curves; (**b**) the pore width. Reprinted with permission from Elsevier 2023 [36].

The authors apologize for any inconvenience caused and state that the scientific conclusions are unaffected. This correction was approved by the Academic Editor. The original publication has also been updated.
